# Prospective Randomized Observational Pilot Trial Evaluating the Effect of Information of Early Childhood Intervention on Stress Levels of Parents of Extremely Low Gestational Age Infants at the NICU

**DOI:** 10.3389/fpubh.2021.684369

**Published:** 2021-09-07

**Authors:** Bernhard Resch, Judith Fröhlich, Katharina Murg, Elisabeth Pichler-Stachl, Claudia Hofbauer-Krug, Ronald Kurz

**Affiliations:** ^1^Research Unit for Neonatal Infectious Diseases and Epidemiology, Medical University of Graz, Graz, Austria; ^2^Division of Neonatology, Department of Pediatrics and Adolescent Medicine, Medical University of Graz, Graz, Austria; ^3^University Course for Interdisciplinary Early Intervention and Family Support, Postgraduate School, Medical University of Graz, Graz, Austria

**Keywords:** neonatal intensive care unit, extremely low gestational age neonates, stress level, early childhood intervention, parental stress scale

## Abstract

It is not known to what extent early information on early childhood intervention (ECI) by ECI professionals reduces or increases stress levels of parents having an extremely preterm infant at the neonatal intensive care unit (NICU). Using an observational pilot study, we gave information on ECI in a randomized matter to parents of an extremely low gestational age newborn (ELGAN) at the chronological age of 3–4 weeks (cases) or not (controls). After informed consent, parents judged the infants at the age of 5–7 weeks with the Parental Stressor Scales: Neonatal Intensive Care Unit [PSS: NICU test has three subscales = “Sights and Sounds” (five items), “Parental Role Alteration” (14 items), and “Look and Behave” (seven items)]. Total scales score and subscales scores were comparable between 13 cases and 13 controls over a study period of 1.5 years. Total scores were 9.32 ± 0.72 in the cases compared to 10.02 ± 0.76 in the controls, (95% CI −6.93 to 4.93). Overall, the cases scored lower in most of the items. Early information on ECI at the NICU was provided to parents with an ELGAN did not result in higher stress levels measured with the PSS: NICU. Whether early information on ECI is a strategy, which might be able to reduce parental stress levels, has to be proven in larger studies.

## Introduction

Birth of an extremely low gestational age newborn (ELGAN) and subsequent stay at the neonatal intensive care unit (NICU) is a frightening experience for parents associated with high-level stress, a number of concerns regarding the survival of the baby, and concerns regarding short- and long-term outcomes. These concerns can have long-lasting negative effects on parental mental health. A tool to measure parental stress having a preterm infant at the NICU is the well-established Parental Stressor Scale (PSS): NICU. A German version used for this pilot study has already been validated too (1–3). Within a certain time window, the PSS:NICU allows measuring parental stress accurately following preterm birth. A homogeneous sample within a defined age of the infants is mandatory for plausible results (4). Over time, parental stress levels change at the NICU (4). Early childhood intervention (ECI) is an interdisciplinary, pedagogic, and holistic approach to infants who either suffer from neurocognitive or motoric impairments or are at high risk for neurodevelopmental impairment. ELGANs are prone to subsequent deficits and need professional care for a longer time than the time at the NICU. Moreover, support like ECI is essential for these infants and young children. In a recent pilot study, we initiated ECI at the NICU and received positive feedback from the parents as a side effect of the study that evaluated the satisfaction of parents getting ECI of different durations (5). Thus, we hypothesized that early contact of the parents of ELGAN with ECI professionals with a piece of standardized information on ECI might be able to reduce parental stress levels measured by the PSS:NICU German at the NICU. We already had performed studies using the PSS:NICU (6, 7). In one study, we assessed maternal stress within 72 h after the birth of a preterm infant who had received antenatal consultation by a neonatologist and compared the stress levels with mothers of matched (by gestational age) controls. We found no substantial influence of antenatal consultation on postnatal stress levels in the mothers of preterm neonates admitted to the NICU (7). A more recent study examined the age dependency of stress in mothers and fathers after preterm birth and subsequent admission of the baby to the NICU. Forty-seven mothers and 47 fathers completed the questionnaire within 72 h after delivery. Parental stress experience after preterm birth tended to be higher in mothers compared to fathers. In mothers, stress levels increased with increasing maternal age, whereas fathers did not show any significant age dependency of stress (6). In the present study, we evaluate parental stress levels markedly later compared to the studies mentioned earlier. We tried to achieve stabilization of stress levels over the first 3–4 weeks of life to better elucidate the presence of a distinct increase or decrease of parental stress levels following consultation on ECI.

This study aimed to evaluate the effect of early information regarding ECI on stress levels of parents of ELGAN at the NICU of the Pediatric Department of the Medical University of Graz, Austria.

## Materials and Methods

Following informed consent parents of an ELGAN (preterm infant below 28 weeks of gestational age) were randomized (https://www.randomizer.at/) by simple random sampling to either receive standardized information on ECI or not at the chronological age of 3–4 weeks of the preterm infant. At the age of 5–7 weeks, parents were asked to answer the PSS: NICU German Version (1). The local ethics committee approved the study (Nr. 31-022 ex 18/19). We planned the sample size to be at least 10 parents in each group. We included some more parents due to possible exclusion reasons including incomplete data or too many “not applicable” answers (predefined more than five items).

### Parental Stressor Scales: Neonatal Intensive Care Unit

The PSS: NICU measures parental stress after preterm birth and admission of their neonate to the NICU. It is a self-report questionnaire, which can be rated on a 5-point Likert scale: 1 = not at all stressful: the experience did not cause you to feel upset, tense, or anxious; 2 = a little stressful; 3 = moderately stressful; 4 = very stressful; and 5 = extremely stressful. Items describing situations that have not been experienced by the parents can also be answered with “not applicable.” The parents should indicate how much experiences were stressful in the three subscales (1):

1) “Sights and Sound” (SS) consisting of five items (e.g., “the presence of monitors and equipment”)
2) “Parental Role Alteration” (PRA) consisting of 14 items (e.g., “being separated from my baby”)
3) “Looks and Behave” (LB) consisting of seven items (e.g., “tubes and equipment on or near my baby”).

**Inclusion criteria**: Informed consent of parents of a preterm infant below 28-week gestation and survival of the first 2 months of life. Mother tongue: German.

**Exclusion criteria**: No informed consent, language difficulties, congenital anomalies, or genetic disorders/chromosomal aberrations.

**Primary outcome:** It was the parental stress level measured with the PSS:NICU at the chronological age of 5–7 weeks of the ELGAN.

**Data collection**: Maternal data included mode of delivery and indication of preterm birth, age, number of pregnancies, number of live-born infants, number of preterm births, the highest level of education, in employment or not before pregnancy, marital status, and for the parent's distance to the hospital (in km). Paternal data included age, the highest level of education, and employment. Neonatal data included gestational age in weeks, birth weight in grams, gender, singleton or multiple, and main neonatal diagnoses.

Statistical analyses were done using Excel© (Microsoft Office, Excel 2013, and CIA© (Statistics with Confidence Interval Analysis, BMJ Books 2000, Bristol, UK, 2nd ed.) for comparison of the PSS: NICU values. Samples were tested for normality with the Shapiro-Wilk test, which is preferred in the case of small numbers. We compared means and SD of total scores, subscales scores, and single items scores by 95% confidence interval (CI) analysis.

## Results

We included 13 cases and 13 controls during a one-and-a-half-year study period. The perinatal data are shown in Table 1. The PSS: NICU was answered within the time window of maximum of 49 days of life in all families.

**Table 1:** Comparison of perinatal data of 13 parents of an ELGAN having received information on ECI (cases) and 13 control parents (controls) without.

	**Cases**	**Controls**
Maternal age (years)	34 (26–41)	31 (22–37)
Paternal age (years)	36 (30–39)	32 (23–41)
Number of pregnancies	1 (0–5)	0.5 (0–6)
Cesarean section	9 (69)	10 (77)
Gestational age (weeks)	25.6 ± 1.4	25.5 ± 1.6
Birth weight (grams)	775 ± 157	721 ± 255
Multiple births	1	0

*Data are given as median (range); mean ± SD; or n (%). ELGAN, extremely low gestational age newborn; ECI, early childhood intervention*.

Parents stated their highest level of education as follows: mothers reported on 8% compulsory education, 22% apprenticeship, 39% higher school certificate, and 39% University degree; fathers reported on 8% compulsory education, 38% apprenticeship, 31% higher school degree, and 23% University degree. About 54% of the couples were married, and all had delivered by cesarean section.

Total scores did not differ between parents with ECI information and those without (see Table 2). Subscale scores tended to be lower in cases (SS items 1, 4, 5; PRA 1, 5–14; LB 1–7) as was the total score. SS items 2 and 3 were comparable between cohorts, and LB items 2–4 tended to be higher in cases. Subscale PRA item 14 (cannot hear my baby cry as other babies do) was lower scored by cases (1.63 ± 0.7 vs. 3.15 ± 1.86; 95% CI 1.43 to 2.57) as was subscale LB item 7 (feeling to have too little time with the baby; 2.08 ± 1.3 vs. 3.5 ± 1.35; 95% CI 0.19 to 1.81).

**Table 2:** Stress levels of parents of an ELGAN assessed with the PSS: NICU questionnaire at the age of 5–7 months: comparison of cases having received standardized information on ECI at the age of about 4 weeks with controls without information.

	**Cases**	**Controls**	**95% CI**
Sights and sounds	2.37 ± 0.90	2.55 ± 0.69	−6.49 to 6.49
Parental role alteration	3.15 ± 0.93	3.41 ± 1.06	−5.35 to 5.35
Looks and behave	3.80 ± 0.79	4.07 ± 0.46	−6.23 to 4.23
Total score	9.32 ± 0.72	10.02 ± 0.76	−6.93 to 4.93

*Data are given as mean ± SD; 95% CI = 95% confidence interval between means. ELGAN, extremely low gestational age newborn; ECI, early childhood intervention; PSS: NICU, parental stress scales: neonatal intensive care unit*.

## Discussion

We could not prove our hypothesis that early contact of parents of ELGANs with ECI professionals at the NICU at the chronological age of 3–4 weeks might reduce parental stress levels measured by the PSS: NICU (German version) at 5–7 weeks of age. We concluded that ECI information did not additionally stress or frighten parents of ELGAN.

Larger cohorts will prove whether this early information might be able to reduce parental stress levels or not. Single PSS: NICU score differences between groups hardly were influenced by the presence or absence of ECI information. Our first results from the Early Bird Study with positive feedback from the parents having had early contact with ECI professionals seem to cope with the presence of an ECI professional rather than the offered ECI information (5). It is always a better feeling to have someone experienced in handling and caring for a premature infant who understands the situation of the parents and empathizes with the parents in the stressful atmosphere of a NICU. Thus, the awareness of ECI for the near future seems to be negligible regarding its influence on parental stress levels.

As already mentioned, we tested markedly later compared to earlier stress studies at our center (6, 7). The goal was to achieve stabilization of stress levels during the first 3–4 weeks of life to better find out a possible influence of ECI consultation. Moreover, we did not differ between mothers and fathers.

The PSS is an internally and over time very reliable questionnaire that relates properly to the general measure of stress. In addition, results are independent of differing parental characteristics, thus suggesting the stability of scale characteristics (8).

Family functioning, socioeconomic status, parent perceptions of infant illness, high-trait anxiety, and available sources of support all contribute to perceptions of stress of parents having an ill neonate or extremely preterm infant admitted to the NICU. These factors potentially contribute to individual differences between maternal and paternal stress awareness (9). Carter et al. (9) investigated sources of stress for mothers and fathers who had an infant admitted to a NICU. In addition, the authors reported on important implications of healthcare professionals assisting parents to adjust to stress in the NICU. It seems important to prepare both the mother and father for alterations that occur in the usual parent–infant relationship. At the same time, healthcare professionals try to involve parents as much as possible in the daily (routine) care of their infant. Parents with elevated trait anxiety are at high risk of experiencing the NICU as stressful, especially regarding the interactions with staff and the preterm appearance and behavior of the infants. Hence, clear communication with the parents and continual discussions and explanations are mandatory (9).

Three decades ago, the efficacy of the developmental intervention in the NICU was evaluated in mothers of preterm infants with low socioeconomic status (10). The intervention group met at least weekly with an infant-development specialist, parents were involved in the structured developmental and behavioral assessment of their infants, and the control group did not. The authors aimed to provide appropriate interactions and environmental stimulation between parents and infants. Interestingly, the infants of the intervention group performed more optimally on the Bayley mental scale (Bayley Scales of Infant Development) at 4 and 8 months of age and the Bayley motor scale at 4 months (10). Other interventions at the NICU including Kangaroo Care or the Newborn Individualized Developmental Care and Assessment Program (NIDCAP) or creative music therapy became increasingly established in the NICUs. Our approach of very early contact of ECI professionals with parents of preterm infants at high risk for developmental disabilities is completely new, and follow-up interviews and further studies have to prove whether this early contact is beneficial or beyond the scope for the parents. Another interventional study compared a nursing interventional program of 30 min of education to routine measures and successfully reduced parental stress at the NICU from 11 days onward (11). A recent meta-analysis on extracted data from 53 studies focusing on parental stress levels at the NICU. Summarizing parental stress related to NICU admission was a worldwide healthcare issue, and in detail, parental role alteration was the greatest source of stress for both mothers and fathers. Mothers had higher stress levels compared to fathers, and interestingly, the characteristics and clinical condition of the baby did not influence parental scores (12).

Limitations of our study have to be mentioned. First, the small study groups and the difficulties to translate the findings of a pilot study to a larger cohort, thus, overall limited generalizability. Second, the long period of one and a half years to collect at least 13 parents in each group, and third, we did not differ between mothers and fathers who are known to have different stress levels at the NICU (13).

In conclusion, early information on ECI did neither reduce nor increase parental stress levels having an ELGAN at the NICU. Thus, implementation of ECI at the NICU was possible without increasing parental stress levels. Further studies might prove the concept of early contact of ECI professionals with parents of ELGANs at the NICU being beneficial to them or not.

## Data Availability Statement

The original contributions presented in the study are included in the article/supplementary material, further inquiries can be directed to the corresponding author/s.

## Ethics Statement

The studies involving human participants were reviewed and approved by Ethics committee of the Medical University of Graz (Nr. 31-022 ex 18/19). The patients/participants provided their written informed consent to participate in this study.

## Author Contributions

BR was responsible for the conceptualization of the study and writing of the final draft. JF and KM were responsible for data curation, administration of the study, and randomization and inclusion of patients. EP-S did the PSS: NICU evaluation and scoring analysis. CH-K was responsible for the organization and training of early childhood intervention professionals and the standardized information in the intervention group. RK reviewed and edited the final manuscript and supervised the correct execution of the study. All authors contributed to the article and approved the submitted version.

## Conflict of Interest

The authors declare that the research was conducted in the absence of any commercial or financial relationships that could be construed as a potential conflict of interest.

## Publisher's Note

All claims expressed in this article are solely those of the authors and do not necessarily represent those of their affiliated organizations, or those of the publisher, the editors and the reviewers. Any product that may be evaluated in this article, or claim that may be made by its manufacturer, is not guaranteed or endorsed by the publisher.
